# Skin Self-Examination Education for Early Detection of Melanoma: A Randomized Controlled Trial of Internet, Workbook, and In-Person Interventions

**DOI:** 10.2196/jmir.2883

**Published:** 2014-01-13

**Authors:** June K Robinson, Rikki Gaber, Brittney Hultgren, Steven Eilers, Hanz Blatt, Jerod Stapleton, Kimberly Mallett, Rob Turrisi, Jenna Duffecy, Mark Begale, Mary Martini, Karl Bilimoria, Jeffrey Wayne

**Affiliations:** ^1^Northwestern UniversityDepartment of DermatologyFeinberg School of MedicineChicago, ILUnited States; ^2^Pennsylvania State UniversityPrevention Research CenterAlcohol and Skin Cancer ProjectsState College, PAUnited States; ^3^Rutgers Cancer Institute of New JerseyRutgers School of Public HealthRutgers, The State University of New JerseyNew Brunswick, NJUnited States; ^4^Center for Behavioral Intervention Technologies (CBITs)Department of Preventive MedicineNorthwestern UniversityChicago, ILUnited States; ^5^Feinberg School of MedicineSurgical Oncology DivisionNorthwestern UniversityChicago, ILUnited States

**Keywords:** melanoma, early detection of cancer, educational techniques, health education, behavioral research, online education, electronic interactive education

## Abstract

**Background:**

Early detection of melanoma improves survival. Since many melanoma patients and their spouses seek the care of a physician after discovering their melanoma, an ongoing study will determine the efficacy of teaching at-risk melanoma patients and their skin check partner how to conduct skin self-examinations (SSEs). Internet-based health behavior interventions have proven efficacious in creating behavior change in patients to better prevent, detect, or cope with their health issues. The efficacy of electronic interactive SSE educational intervention provided on a tablet device has not previously been determined.

**Objective:**

The electronic interactive educational intervention was created to develop a scalable, effective intervention to enhance performance and accuracy of SSE among those at-risk to develop melanoma. The intervention in the office was conducted using one of the following three methods: (1) in-person through a facilitator, (2) with a paper workbook, or (3) with a tablet device used in the clinical office. Differences related to method of delivery were elucidated by having the melanoma patient and their skin check partner provide a self-report of their confidence in performing SSE and take a knowledge-based test immediately after receiving the intervention.

**Methods:**

The three interventions used 9 of the 26 behavioral change techniques defined by Abraham and Michie to promote planning of monthly SSE, encourage performing SSE, and reinforce self-efficacy by praising correct responses to knowledge-based decision making and offering helpful suggestions to improve performance. In creating the electronic interactive SSE educational intervention, the educational content was taken directly from both the scripted in-person presentation delivered with Microsoft PowerPoint by a trained facilitator and the paper workbook training arms of the study. Enrollment totaled 500 pairs (melanoma patient and their SSE partner) with randomization of 165 pairs to the in-person, 165 pairs to the workbook, and 70 pairs to electronic interactive SSE educational intervention.

**Results:**

The demographic survey data showed no significant mean differences between groups in age, education, or income. The tablet usability survey given to the first 30 tablet pairs found that, overall, participants found the electronic interactive intervention easy to use and that the video of the doctor-patient-partner dialogue accompanying the dermatologist’s examination was particularly helpful in understanding what they were asked to do for the study. The interactive group proved to be just as good as the workbook group in self-confidence of scoring moles, and just as good as both the workbook and the in-person intervention groups in self-confidence of monitoring their moles. While the in-person intervention performed significantly better on a skill-based quiz, the electronic interactive group performed significantly better than the workbook group. The electronic interactive and in-person interventions were more efficient (30 minutes), while the workbook took longer (45 minutes).

**Conclusions:**

This study suggests that an electronic interactive intervention can deliver skills training comparable to other training methods, and the experience can be accommodated during the customary outpatient office visit with the physician. Further testing of the electronic interactive intervention’s role in the anxiety of the pair and pair-discovered melanomas upon self-screening will elucidate the impact of these tools on outcomes in at-risk patient populations.

**Trial Registration:**

ClinicalTrials.gov NCT01013844; http://clinicaltrials.gov/show/NCT01013844 (Archived by WebCite at http://www.webcitation.org/6LvGGSTKK).

## Introduction

### Background

Early detection of melanoma improves survival. In those diagnosed with Stage IA melanoma, the survival rate 10 years after diagnosis was estimated at higher than 95%, which declined to less than 60% when diagnosis is delayed (Stage IIB, C). Previous research has found that melanoma patients and their spouses often discover the melanoma and then seek the care of a physician [1,2]. Any enhanced surveillance for melanoma patients has great potential to detect future melanomas in their earliest stages when treatment prognosis is optimal. Robinson et al found that melanoma patients and their skin check partners can learn the skin self-examination (SSE) skills and that these skills can be improved by routine practice [3,4]. The most appropriate delivery method for the educational and skills training intervention remains unclear; thus, we present an interim analysis of an ongoing clinical trial following subjects at 4-month intervals for 2 years to determine the efficacy of teaching at-risk melanoma patients and their skin check partners how to conduct deliberate skin self-examinations.

The purpose of this study is to develop and evaluate an electronic interactive educational program that provides SSE skills training for at-risk melanoma patients and their partners in an effort to help them enhance their participation and performance of SSE. Internet interventions designed to educate patients about health promotion and personal health care are becoming widely available. A meta-analysis of 85 different Internet intervention programs that promoted health behavior change found that those grounded in the theory of planned behavior were among the most efficacious programs [5]. Interventions modeled after the theory of planned behavior often include content related to modeling skills, prevention planning, goal setting, action planning, and feedback on performance [6]. When this theory is applied to the health care education of patients at risk for developing a second melanoma, the education and skills training in performing SSE may prove extremely beneficial in improving the survival of the patient [7].

This study compares different methods of educating both patients with a history of melanoma and their skin check partners about the need and proper techniques to conduct skin examinations and identify clinically suspicious moles. This study used three educational interventions with identical education components delivered in different formats: (1) in-person PowerPoint presentation delivery by a trained facilitator, (2) a self-guided paper workbook [8], and (3) an electronic interactive intervention delivered on a tablet personal computer (PC). We compared the pairs’ confidence in performing SSE and knowledge-based performance immediately after receiving the interventions delivered in-person through a facilitator, with a paper workbook, or a tablet device used in the clinical office in order to elucidate differences related to the method of delivery of the intervention.

### Objective

An electronic interactive educational program was created to develop a scalable, effective intervention to enhance performance and accuracy of SSE among those at risk to develop melanoma.

## Methods

### Design

Educational content delivered in each intervention included five elements: modeling of skills, prevention planning, goal setting, action planning, and feedback on performance, which are crucial in creating behavior and attitude change [5]. The three interventions used the following 9 of the 26 behavioral change techniques defined by Abraham and Michie to enhance patient performance and confidence outcomes [9]: (1) information given about melanoma, ability to find it early if the person is trained and actively looking, (2) information about the spread of cancer to other organs, surgery required, mortality statistics, (3) encourage pair to act within 2 weeks of the skills training session, (4) praise selection of correct scores for border, color, and diameter and encourage to keep trying, (5) trained research assistants or physician demonstrated how to correctly perform the behavior, (6) set a goal of checking five moles together each month for the first 4 months, (7) pair uses a body map and scorecard to record their findings, (8) show partners how to check for moles in places patients cannot see themselves, and (9) 4-month follow-up appointments to encourage continued SSE (behavior change). Five moles were selected as the goal to examine each month because in our previous work the majority of melanoma patients had five moles that the physician determined needed to be followed for change [3,8].

The inclusion criteria were having a history of Stage 0 to IIB melanoma and being at least 6 weeks after surgical treatment of melanoma, able to see to read a newspaper, fluent in English, age 21-80, and having significant other person (spouse, partner, close relative) who was willing to participate in the research. Exclusion criteria were a history of Stage III or greater melanoma; ocular, genital, and oral melanoma; being overburdened with comorbid disease; unable to see to read a newspaper; not fluent in English; unable to participate in conversation at a sixth grade language level due to cognitive impairment; and did not have a significant other person (spouse, partner, close relative) who was willing to participate in the research. The Institutional Review Board of Northwestern University approved the study.

**Figure 1:**
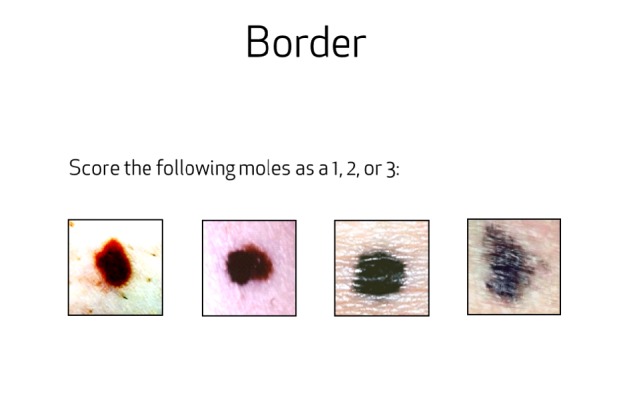
Interactive education on borders of moles.

**Figure 2:**
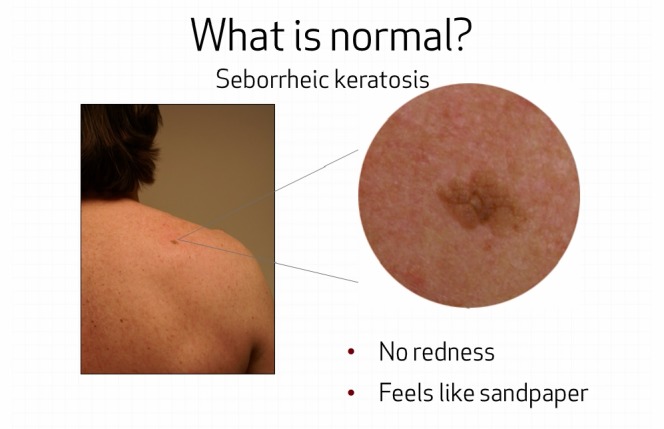
Teaching about normal growths.

Content across all three interventions was controlled for and remained as similar as possible under the restraints of different delivery systems. With respect to delivery, the electronic interactive educational program was delivered on two tablets—one to each member of the pair simultaneously with earphones. The tablet was a Samsung CE 0168 (US $249.00) touch screen device held in the person’s hand or set upright on the table for viewing. The tablet PC was used to deliver the intervention in the ambulatory care setting but was not given to the pair to take home. In order to control for dosage effects when comparing with the in-person education, the electronic interactive education was also withheld from the intervention group so it could not be viewed at home. Due to the mode of delivery, the electronic interactive program contained more uniformity in the interactive components than the in-person intervention. While content itself remained similar, the electronic interactive program had more multidimensional learning tools, such as a narrated video presentation and animated graphics to enhance the educational experience. In all three interventions, when learning about scoring the border, color, and diameter of moles, patients and their partners were quizzed on the material and received feedback as to whether they answered correctly (see Figure 1). Finally, pairs also learned about two benign lesions commonly found on skin, seborrheic keratoses and cherry angiomas, and how to differentiate them from moles that may be suspicious (see Figure 2).

**Figure 3:**
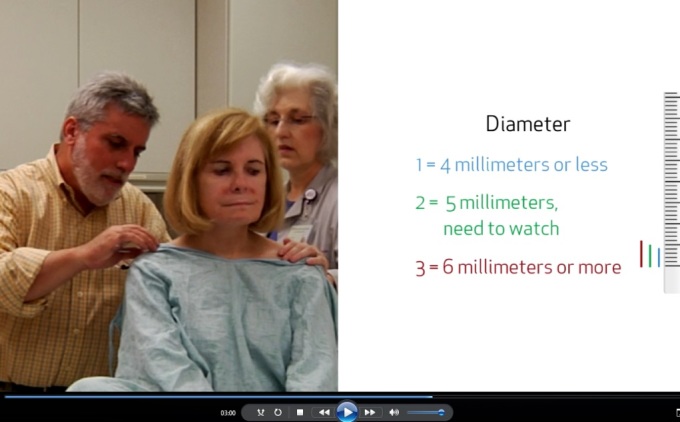
Video component of education program.

One of the modules in the intervention asked pairs to begin their SSE by picking five moles on the patient’s body to score and watch for change over the initial 4 months. This action planning was further encouraged by the use of a “skin diary of a body map and a scorecard” to record their observations of their chosen moles, with the goal of having the pair make a commitment to conduct the skin examination each month. In the electronic interactive intervention, use of the skin diary was modeled. Pairs participating in all three interventions were also told that they would review the moles they chose to observe with the doctor at the 4-month visit. In the electronic interactive intervention, the doctor-patient-partner interaction that occurred at the 4-month follow-up visit was demonstrated in a video as a learning experience (see Figure 3). This representation of the demonstration of scoring the border, color, and diameter of the mole with the doctor during the appointment allowed the pair to see what was expected of them at the visit, as well as what they could expect to learn from the doctor during the visit.

### Measures

**Figure 4:**
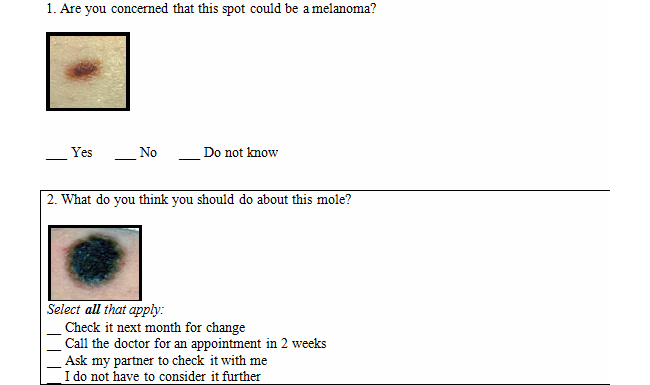
Sample questions from the skills quiz.

Participants completed a pre-intervention survey that included demographic questions as well as questions pertaining to their use of Internet and mobile phone technology (see Table 1). After the survey, the pairs were randomized to one of the four arms (three interventions and customary education provided in clinical practice, which was the control condition). Pairs randomized to the control condition did not receive the measures reported in this interim analysis; therefore, there is no presentation of data for the control group. Each individual in the pair completed an evaluation of the intervention consisting of 22 items with a 5-point Likert scale assessing the following domains: clarity of explanation of the ABCDE rule, scoring the three features (border, color, and diameter) and using body maps to locate the mole, confidence in performing SSE, and ease of understanding the content (see Table 2). After participating in the education, the participating pairs were given a skills quiz consisting of life-size photographs of five pigmented lesions printed in color with questions. The pair was asked to discuss and come to agreement on their response (Figure 4). The first 30 pairs in the electronic interactive intervention group also completed a further Tablet Usability survey giving their opinions on the intervention. The Tablet Usability survey was administered to six groups of five pairs, and analysis was performed after each set of five pairs to identify changes that needed to be made to the tablet presentation design, but not content.

**Table 1:** Demographic information^a^.

Characteristics		Workbook (n=165), n (%)	Electronic interactive (n=70), n (%)	In-person (n=165), n (%)
**Education**				
	No high school	0 (0)	0 (0)	1 (0.6)
	Some high school	2 (1.2)	0 (0)	1 (0.6)
	High school graduate	4 (2.4)	3 (4.3)	9(5.4)
	Some post-high school education	19 (11.5)	5(7.2)	27(15.4)
	College graduate	76 (46.1)	19(27.1)	63(38.2)
	Graduate degree	57(34.5)	26(37.1)	64(38.8)
	Unanswered	2 (1.2)	0(0)	2 (1.2)
**Income (USD)**				
	<$10,000	1(0.6)	1(1.4)	2 (1.2)
	$10,000-$19,999	1 (0.6)	1(1.4)	1 (0.6)
	$20,000-$34,999	8(4.8)	1(1.4)	8 (4.8)
	$35,000-$50,999	8(4.8)	2(2.8)	12(7.3)
	$51,000-$100,000	45 (27.3)	26(37.1)	43(26.1)
	>$100,000	94(57.0)	22(31.4)	97(58.8)
	Unanswered	3 (1.8)	0 (0)	4 (2.4)
Age (years), mean (SD)		55.19 (14.12)	55.19 (14.12)	54.70 (14.84)

^a^No significant mean differences between groups in age, education, or income.

**Table 2:** Skills performance^a^.

	Workbook (n=165)	Electronic interactive (n=70)	In-person (n=165)
Mean (SD)	3.02 (0.82)^b^	3.25 (0.68)	3.47 (0.69)^c^

^a^1-way analysis of variance showed a significant difference in means (*P*<.001); *F*_10.31_=14.98.

^b^Significantly different from In-person group.

^c^Significantly different from Workbook group.

### Recruitment

**Figure 5:**
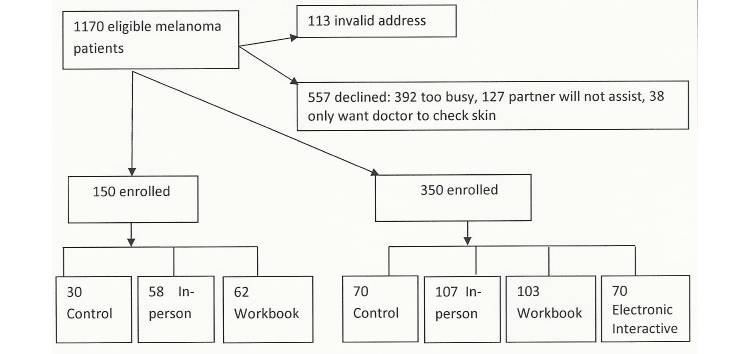
Flow chart of study recruitment.

Pairs were recruited through the Northwestern Memorial Faculty Foundation Department of Dermatology at Northwestern University Feinberg School of Medicine in Chicago, IL. Enrollment totaled 500 pairs with randomization of 165 pairs to the in-person, 165 pairs to the workbook, and 70 pairs to the electronic interactive pilot intervention arms, with 100 pairs to the control arm or education as usually performed (see Figure 5). While accruing the initial 150 pairs, we noticed that the pairs reported reading the workbook was a burden; therefore, it was decided to explore the use of an electronic interactive intervention delivered with a tablet during an office visit as another easily disseminated form of the intervention. The sample randomized to the fourth arm (electronic interactive intervention) is smaller than the other two interventions because it was not intended to be subjected to factor analysis, for example, influence of dyadic relationship. Rather, the fourth arm (electronic interactive intervention) will be compared with the others solely on the basis of performance of SSE and accuracy of SSE in comparison with the dermatologist’s skin examination over the 2 years.

## Results

The demographic survey data showed no significant mean differences between groups in age, education, or income. In all groups, the majority of individuals had a college degree or higher and had an income of US $51,000 or higher (see Table 1). While over half of study participants randomized to the electronic interactive intervention (39/70, 55%) owned a tablet, 72% (50/70) of study participants had never used their tablet for interactive educational materials. The most common use of the tablet was to read books and email. This research experience with the electronic interactive intervention was a new use of the tablet for most participants.

The Tablet Usability survey given to the first 30 electronic interactive intervention pairs found that, overall, participants found the electronic interactive intervention easy to use and that the video of the doctor-patient-partner dialogue accompanying the dermatologist’s examination was particularly helpful in understanding what they would be doing. The initial 15 pairs experienced technical issues in using the tablet, for example, delay in loading images and failure to load chapters without rebooting the device. These technical concerns were resolved before continuing with the rest of the participating pairs.

The duration of the pairs’ exposure to the intervention was about 30 minutes for both the in-person and the electronic interactive interventions. Those receiving the workbook intervention spent about 45 minutes reading it. Knowledge of SSE, using the scoring system, and body map diaries were similar across all three interventions. Clarity of the explanation of evolution and scoring in the in-person group were significantly different from the workbook and electronic interactive groups (see Table 3). While the in-person group reported a slightly higher score in the ease of understanding, it was not significantly different from the electronic interactive group, and both the in-person and electronic interactive groups reported a significantly greater ease in understanding than the workbook group. There was a significant difference in the self-efficacy of the pairs receiving the three interventions. The in-person group had significantly higher reports of confidence to monitor and score their moles compared to the workbook group, whereas the electronic interactive group did not significantly differ from either the workbook groups on scoring the moles.

The skills quiz assesses the pairs’ use of the ABCDE scoring rules to make correct decisions about five pigmented lesion examples. Two types of questions were used. There were five questions of the first type with each having one correct answer (n=5) and two questions of the second type with one question having two correct responses and one having one correct response (n=3). A higher score indicated better decisions (range 0-5) (see Figure 4). The results of the skills quiz across the three intervention groups showed that the pairs in the in-person group scored significantly higher than the pairs in the workbook group. However, the electronic interactive intervention group scored significantly higher than their counterparts in the workbook group (1-way analysis of variance showed a significant difference in means, *P*=.01) (see Table 2).

**Table 3:** Significant differences in means among interventions based on 1-way analysis of variance^a,b^. Questions were evaluated using a 5-point Likert scale coded between –2 [strongly disagree] and +2 [strongly agree].

Item	Workbook (n=165), mean (SD)	Electronic interactive (n=70), mean (SD)	In-person (n=165), mean (SD)	*F* (df)
Evolution was explained clearly with useful examples	1.80 (0.40)^c^	1.81 (0.40)^c^	1.96 (0.23)^a,b^	10.23 (2.63)
Scoring was explained clearly with useful examples	1.60 (0.40)^c^	1.68 (0.55)^c^	1.94 (0.26)^a,b^	19.27 (6.91)
I feel that I am now better able to monitor my moles over time	1.53 (0.58)^c^	1.60 (0.49)	1.78 (0.45)^a^	10.09 (4.72)
I feel that I am now better able to score moles accurately	1.41 (0.66)^c^	1.47 (0.58)^c^	1.70 (0.51)^a,b^	10.45 (5.14)
Overall, the information was easy to understand	1.62 (0.57)^c^	1.72 (0.50)	1.79 (0.46)^a^	4.43 (1.26)

^a^Significantly different from Workbook group.

^b^Significantly different from Internet group.

^c^Significantly different from In-person group.

## Discussion

### Principal Findings

In this study, the immediate post-intervention performance skills of participants in the electronic interactive group were no different than those in the in-person education group, but higher than the workbook group. This skills test performance suggested an electronic interactive intervention can be more effective than passively reading information and just as effective as an in-person intervention. The advantages of the electronic interactive intervention were the consistent delivery and the reported understanding of the SSE task, especially evolution. It is possible that the virtual placement of the physician in the videography provided a more personal experience for the patient by modeling the expected interaction between the doctor and the pair. Thus, the electronic interactive intervention may motivate the pair to practice SSE in order to form opinions and questions to discuss with the doctor in a way that cannot be achieved via workbook or the in-person intervention. The electronic interactive intervention and the in-person intervention require less time from the participant (30 minutes) than reading the workbook (45 minutes). In addition to the workbook being a burden to the participant, the electronic interactive intervention will be a more efficient use of personnel and space in the office than an in-person intervention. Overall, this study suggests that an electronic interactive intervention can deliver skills training comparable to other training methods and the experience can be accommodated during the customary outpatient office visit with the physician.

The electronic interactive intervention was effective in teaching skills to patients and their skin check partners. Even pairs who had no prior experience with receiving educational information from a tablet PC or no prior experience with a tablet PC readily began using the device. Thus, it was not essential that the user be familiar with using the technology platform to benefit from education materials on these platforms. This allowed the electronic interactive intervention to be effective across a range of socioeconomic classes or among elderly patients with less experience with technology. However, because the intervention required a secure Internet connection, the feasibility of using this tablet intervention to supplement the usual patient education depended on whether the doctor’s office was able to provide Internet access. To help overcome this obstacle, our technology team determined a method to load the program into the tablet’s cache, where it was stored and accessible without an Internet connection. An obstacle to this method is that it requires significant coordination with respect to the technology team, the tablet, and the office using the device, as each update to the tablet and other random events can break down the consistent experience observed in the Internet-based format. While this cache-loaded format was not used in the study, the experience is exactly the same as the Internet-based format.

The electronic interactive intervention may prove to be a more consistent method of teaching patients and their skin-check partner how to conduct monthly SSEs than having a conversation with a health care provider and could be provided at less cost than delivery by a health care provider. Further, the electronic interactive intervention may be made available via the Internet to a wider audience than may be seen by a health care provider. In the future, a technologically more sophisticated and interactive Internet-based intervention may approach that of a face-to-face intervention in promoting self-efficacy and clarity of explanation of content. A caveat is that much of the cost associated with the Internet intervention is incurred at the design and development stage rather than in delivering the intervention to the individual patient. Further, the cost per patient or per life saved decreases significantly when such a program is scaled to a population, as can be accomplished via the Internet.

### Limitations

Since the fiscal commitment to developing the electronic interactive intervention was small, the electronic interactive intervention had limited interactivity and personalization. The sample size was limited to one institution and the population was of a slightly higher socioeconomic status (income of US $51,000 and higher) than the national income average (US $42,979), which may limit generalizability of our findings, especially in regards to prior experience with the tablet technology [10]. In the future, this pilot electronic interactive intervention will be redesigned to be more interactive, which is expected to improve the pairs’ self-efficacy such that the intervention will be comparable with the in-person education.

As the study has not finished the 2-year period of follow-up with the participating pairs, conclusions cannot be made about the accuracy of SSE as performed by the pairs in comparison with the diagnosis of the pigmented lesion by a dermatologist.

### Conclusions

This study suggested that an electronic interactive intervention can deliver skills training comparable to other training methods and the experience can be accommodated during the customary outpatient office visit with the physician. Reducing the burden on the health care provider’s time to educate patients in person may make it possible to reach large numbers of people at risk for melanoma. As such, this method can be used as the primary intervention, or as an easily accessible Internet-based approach to reinforce education provided in the office. The potential for dissemination of this information to people who are at risk of developing melanoma could greatly improve the rate of early detection of melanoma, as well as reduce patient anxiety about reoccurrence as the patient feels more confident and competent at performing SSE.

## Abbreviations

PC: personal computer
SSE: skin self-examination

## Multimedia Appendix 1

CONSORT-EHEALTH checklist V1.6.2 [11].
