# Supplementary material for: Skin Self-Examination Education for Early Detection of Melanoma: A Randomized Controlled Trial of Internet, Workbook, and In-Person Interventions
Source: J Med Internet Res. 2014 Jan 13;16(1):e7. doi: 10.2196/jmir.2883 (PMC3906663; doi:10.2196/jmir.2883)
Supplement: Supplementary file 1 [file jmir_v16i1e7_app1.pdf]

CONSORT-EHEALTH Checklist V1.6.2 Report

(based on CONSORT-EHEALTH V1.6), available at [http://tinyurl.com/consort-ehealth-v1-6].

2883

Date completed

8/15/2013 17:28:41

by

Rikki Gaber

Skin self-examination education for early detection of melanoma: A randomized control trial of Internet, workbook and in-person interventions

TITLE

1a-i) Identify the mode of delivery in the title

"Internet intervention"

1a-ii) Non-web-based components or important co-interventions in title

"in-person"
"workbook"

1a-iii) Primary condition or target group in the title

"Skin self-examination education for early detection of melanoma"

ABSTRACT

1b-i) Key features/functionalities/components of the intervention and comparator in the METHODS section of the ABSTRACT

"The three interventions used nine of the 26 behavioral change techniques defined by Abraham and Michie to promote planning of monthly SSE, encourage performing SSE and reinforce self-efficacy by praising correct responses to knowledge based decision making and offering helpful suggestions to improve performance [8]. In creating the Internet intervention, the educational content was taken directly from the scripted in-person (face-to-face) PowerPoint presentation delivered by a trained facilitator and workbook training arms of the study."

1b-ii) Level of human involvement in the METHODS section of the ABSTRACT

1b-iii) Open vs. closed, web-based (self-assessment) vs. face-to-face assessments in the METHODS section of the ABSTRACT

The outcomes were self-assessed through questionnaires as well as a skill quiz.

1b-iv) RESULTS section in abstract must contain use data

"There was a significant difference in the self-efficacy of the pairs receiving the three interventions with the Internet group being more confident than the workbook group, but not as confident as the pairs in the in-person intervention (Chi-square, df= 10.427, P= .003). In the skill quiz, the Internet intervention group scored significantly higher than their counterparts in the workbook group (1-way analysis of variance showed a significant difference in means, P=.01)."

1b-v) CONCLUSIONS/DISCUSSION in abstract for negative trials

"This study suggests that an Internet intervention can deliver skills training comparable to other training methods and the experience can be accommodated during the customary outpatient office visit with the physician. The potential for dissemination of this information to people who are at risk of developing melanoma could greatly improve the rate of early detection of the skin cancer, as well as reduce patient anxiety about reoccurrence as the patient feels more confident and competent at performing self-skin examination."

INTRODUCTION

2a-i) Problem and the type of system/solution

"Melanoma is a serious skin cancer that can metastasize. In those diagnosed with Stage IA melanoma, the survival rate 10 years after diagnosis was estimated as higher than 95% and declined to less than 60% when diagnosis occurred later (Stage IIB, C). People who are diagnosed later rapidly progress to the advanced stage of the disease with metastasis to internal organs (Stage IV). However, if found early melanoma can be successfully treated. Previous research has found that melanoma patients who conduct deliberate skin self-examinations (SSE's) are more likely to survive than those who discover melanoma incidentally [4, 5]. Therefore, enhanced surveillance for melanoma patients has the greatest potential to detect future melanomas in their earliest stages where treatment prognosis is optimal."

2a-ii) Scientific background, rationale: What is known about the (type of) system

"A meta-analysis of 85 different Internet interventions measured the efficacy of these programs in promoting health behavior change. The study found that the Internet interventions grounded in the theory of planned behavior tended to have significant effects on the behavior of participants of these studies [1]"

METHODS

3a) CONSORT: Description of trial design (such as parallel, factorial) including allocation ratio

An Internet-based educational program was created to develop a scalable, effective intervention to enhance performance and accuracy of SSE among those at-risk to develop melanoma. Its efficacy was compared to that of an in-person intervention and a workbook intervention.

3b) CONSORT: Important changes to methods after trial commencement (such as eligibility criteria), with reasons

No changes were made after the commencement of the pilot arm of the Internet intervention.

3b-i) Bug fixes, Downtimes, Content Changes

"The initial 15 pairs experienced technical issues in using the tablet, e.g. delay in loading images and failure to load chapters without rebooting the device. These technical concerns were resolved before continuing with the rest of the participating pairs."

4a) CONSORT: Eligibility criteria for participants

"Eligibility criteria included: the patient must have a history of melanoma (Stage 0 -2B), be able to read and understand English, be between 18-80 years old, and have a partner who can make all of the study visits."

4a-i) Computer / Internet literacy

4a-ii) Open vs. closed, web-based vs. face-to-face assessments:

"Pairs were recruited through the Northwestern Memorial Faculty Foundation Department of Dermatology clinic at Northwestern University Feinberg School of Medicine in Chicago, IL."

The following assessments were completed on paper, but the coordinator was not in the room with the pair as they completed the assessments. Thus, not online assessments, but also not face-to-face assessments. "At the baseline visit prior to randomization, participants were asked to complete a survey that included demographic questions as well as questions pertaining to their use of Internet and mobile phone technology (Table 2). After the survey, the pairs were randomized to one of the four arms (3 interventions and standard of care, which was the control condition). Each individual in the pair completed an evaluation of the intervention consisting of 22 items with a 5–point Likert scale assessing the following domains: clarity of explanation of knowledge about the ABCDE rule, scoring the three features (border, color and diameter) and using body maps to locate the mole; confidence in performing SSE, and ease of understanding the content (Table 3). After participating in the education, the participating pairs were given a skills quiz consisting of life-size photographs of 5 pigmented lesions printed in color with questions."

4a-iii) Information giving during recruitment

Not relevant to the purpose of our paper.

4b) CONSORT: Settings and locations where the data were collected

"Pairs were recruited through the Northwestern Memorial Faculty Foundation Department of Dermatology at Northwestern University Feinberg School of Medicine in Chicago, IL." The data was also collected at NU.

4b-i) Report if outcomes were (self-)assessed through online questionnaires

The self-assessments were done on paper, not online.

4b-ii) Report how institutional affiliations are displayed

The institutional affiliations were not displayed on the interventions other than the cover page in the bottom corner.

5) CONSORT: Describe the interventions for each group with sufficient details to allow replication, including how and when they were actually administered

5-i) Mention names, credential, affiliations of the developers, sponsors, and owners

This is not applicable for our paper because the study has not finished yet.

5-ii) Describe the history/development process

"In creating the Internet intervention, the educational content was taken directly from the scripted in-person (face-to-face) PowerPoint presentation delivered by a trained facilitator and workbook training arms of the study. Thus, every pair of participants randomized to an intervention received the same information. The Internet program delivered to each member of the pair with a tablet personal computer (PC) held in the person's hands contained more uniformity in the interactive components than the in-person intervention."

5-iii) Revisions and updating

The intervention did not go through any major revisions during the time of the Internet intervention study so this was not relevant to mention in the paper.

5-iv) Quality assurance methods

5-v) Ensure replicability by publishing the source code, and/or providing screenshots/screen-capture video, and/or providing flowcharts of the algorithms used

5-vi) Digital preservation

We did not mention preservation of the application URL because it will not be published for a year or so.

5-vii) Access

"The participants in the Internet intervention arm were each given a tablet that was already wireless connected to the Internet with the program ready to start. They were asked to press start and follow the instructions.They completed this intervention in the research office, alone as a pair."

5-viii) Mode of delivery, features/functionalities/components of the intervention and comparator, and the theoretical framework

"The Internet program had more opportunities for interaction than the workbook intervention. For example, the Internet program had a narrated video presentation, and animated graphics to enhance the educational experience. In all three interventions, when learning about scoring the border, color, and diameter of moles, patients and their partners were quizzed on the material, and received feedback as to whether they answered correctly (Figure 1). Pairs also learned about two benign lesions commonly found on skin, seborrheic keratoses and cherry angiomas, and how to differentiate them from moles that may be suspicious (Figure 2).
The three interventions used nine of the 26 behavioral change techniques defined by Abraham and Michie to promote planning of monthly SSE, encourage performing SSE and reinforce self-efficacy by praising correct responses to knowledge based decision making and offering helpful suggestions to improve performance [8] (Table 1). A meta-analysis of Internet interventions found that the most effective behavioral change techniques used in the interventions included the following five items: modeling, prevention planning, goal setting, action planning and feedback on performance [1]. The educational content delivered in the three styles of interventions included these five elements crucial in creating behavior and attitude change. One of the modules in the intervention asked pairs to begin their SSE by picking 5 moles on the patient's body to score and watch for change over the initial four months. This action planning was further encouraged by the use of a "skin diary of a body map and a scorecard" to record their observations of their chosen moles, with the goal of having the pair make a commitment to conduct the skin examination each month. In the Internet intervention, use of the skin diary was modeled. Pairs participating in all three interventions were also told that they will review the moles they chose to observe with the doctor at the 4 month visit. In the Internet intervention, the doctor- patient- partner interaction that occurred at the 4-month visit was demonstrated in a video as a learning experience (Figure 3). This representation of the demonstration of scoring the border, color and diameter of the mole with the doctor during the appointment allowed the pair to see what was expected of them at the visit, as well as what they could expect to learn from the doctor during the visit."

"A meta-analysis of 85 different Internet interventions measured the efficacy of these programs in promoting health behavior change. The study found that the Internet interventions grounded in the theory of planned behavior tended to have significant effects on the behavior of participants of these studies [1]. The theory of planned behavior links the idea that the attitude one holds regarding their own behavior and their sense of control factor into a person's intentions and motivations [2]. When this theory is applied to the healthcare education of patients at risk for developing a second melanoma, the education and skills training in performing skin self-examination (SSE) may prove extremely beneficial in improving the survival of the patient [3]."

5-ix) Describe use parameters

5-x) Clarify the level of human involvement

5-xi) Report any prompts/reminders used

No prompts were given, as this study is just looking at baseline measures. No reminders were given at the baseline visits.

5-xii) Describe any co-interventions (incl. training/support)

Co-interventions not relevant to the study. This was a stand-alone intervention.

6a) CONSORT: Completely defined pre-specified primary and secondary outcome measures, including how and when they were assessed
Each individual in the pair completed an evaluation of the intervention consisting of 22 items with a 5–point Likert scale assessing the following domains: clarity of explanation of knowledge about the ABCDE rule, scoring the three features (border, color and diameter) and using body maps to locate the mole; confidence in performing SSE, and ease of understanding the content (Table 3). After participating in the education, the participating pairs were given a skills quiz consisting of life-size photographs of 5 pigmented lesions printed in color with questions.
6a-i) Online questionnaires: describe if they were validated for online use and apply CHERRIES items to describe how the questionnaires were designed/deployed

6a-ii) Describe whether and how "use" (including intensity of use/dosage) was defined/measured/monitored

6a-iii) Describe whether, how, and when qualitative feedback from participants was obtained

6b) CONSORT: Any changes to trial outcomes after the trial commenced, with reasons
No changes, not relevant to our paper.
7a) CONSORT: How sample size was determined
7a-i) Describe whether and how expected attrition was taken into account when calculating the sample size
Not relevant to the purpose of our paper.
7b) CONSORT: When applicable, explanation of any interim analyses and stopping guidelines
This was not applicable to the paper.
8a) CONSORT: Method used to generate the random allocation sequence
"Enrollment totaled 500 pairs with randomization of 165 pairs to the in-person, 165 pairs to the workbook; and 70 pairs to the Internet pilot intervention arms. In addition, 100 pairs were randomized to the control or education as usually performed. This was a computer-generated randomization process and was implemented through random assignment to one of the groups; the assignment was delivered by a research coordinator in an envelope at the pair's study visit"
8b) CONSORT: Type of randomisation; details of any restriction (such as blocking and block size)
This was not included in the paper.
9) CONSORT: Mechanism used to implement the random allocation sequence (such as sequentially numbered containers), describing any steps taken to conceal the sequence until interventions were assigned
"This was a computer-generated randomization process and was implemented through random assignment to one of the groups; the assignment was delivered by a research coordinator in an envelope at the pair's study visit"
10) CONSORT: Who generated the random allocation sequence, who enrolled participants, and who assigned participants to interventions
"...the assignment was delivered by a research coordinator in an envelope at the pair's study visit"
11a) CONSORT: Blinding - If done, who was blinded after assignment to interventions (for example, participants, care providers, those assessing outcomes) and how
11a-i) Specify who was blinded, and who wasn't
The PI (doctor) was blinded as to what group each participant was in. The research coordinators were not blinded. The pairs were not blinded.
11a-ii) Discuss e.g., whether participants knew which intervention was the "intervention of interest" and which one was the "comparator"
Not relevant to the purpose of our paper.
11b) CONSORT: If relevant, description of the similarity of interventions
"In creating the Internet intervention, the educational content was taken directly from the scripted in-person (face-to-face) PowerPoint presentation delivered by a trained facilitator and workbook training arms of the study. Thus, every pair of participants randomized to an intervention received the same information."
12a) CONSORT: Statistical methods used to compare groups for primary and secondary outcomes
P-values and chi-squared test.
12a-i) Imputation techniques to deal with attrition / missing values
Analyses not performed for this paper.
12b) CONSORT: Methods for additional analyses, such as subgroup analyses and adjusted analyses
Additional analyses not performed for this paper.

RESULTS

13a) CONSORT: For each group, the numbers of participants who were randomly assigned, received intended treatment, and were analysed for the primary outcome
"The recruitment goals were met; N = 500; 165 in each the in-person and workbook groups, 100 in the control group and 70 in the Internet group."
13b) CONSORT: For each group, losses and exclusions after randomisation, together with reasons
The study is looking at the first baseline visit, where there were no losses or exclusions.
13b-i) Attrition diagram
Not relevant to our study design or what our paper is looking at.
14a) CONSORT: Dates defining the periods of recruitment and follow-up
"These pairs were enrolled from August 2011 through April 2013."
14a-i) Indicate if critical "secular events" fell into the study period
Not relevant to the paper.
14b) CONSORT: Why the trial ended or was stopped (early)
Not applicable to the study. Did not stop early.
15) CONSORT: A table showing baseline demographic and clinical characteristics for each group
Not relevant to the aim of our paper. Table 1 shows demographics but not clinical characteristics.
15-i) Report demographics associated with digital divide issues
Not relevant. There were no demographic differences.
16a) CONSORT: For each group, number of participants (denominator) included in each analysis and whether the analysis was by original assigned groups
16-i) Report multiple "denominators" and provide definitions
"The recruitment goals were met; N = 500; 165 in each the in-person and workbook groups, 100 in the control group and 70 in the Internet group."
16-ii) Primary analysis should be intent-to-treat

17a) CONSORT: For each primary and secondary outcome, results for each group, and the estimated effect size and its precision (such as 95% confidence interval)
Analyses not performed for this paper.
17a-i) Presentation of process outcomes such as metrics of use and intensity of use
Analyses not performed for this paper.
17b) CONSORT: For binary outcomes, presentation of both absolute and relative effect sizes is recommended
Analyses not performed for this paper.
18) CONSORT: Results of any other analyses performed, including subgroup analyses and adjusted analyses, distinguishing pre-specified from exploratory
Not performed for this paper.
18-i) Subgroup analysis of comparing only users
Not performed for this paper.
19) CONSORT: All important harms or unintended effects in each group
Not relevant to the study because it is educational; no unintended effects or harms were reported.
19-i) Include privacy breaches, technical problems
"The initial 15 pairs experienced technical issues in using the tablet, e.g. delay in loading images and failure to load chapters without rebooting the device. These technical concerns were resolved before continuing with the rest of the participating pairs"
19-ii) Include qualitative feedback from participants or observations from staff/researchers
"The Tablet Usability survey given to the first 30 tablet pairs found that overall, participants found the Internet intervention easy to use and personal, and that the video of the doctor-patient- partner dialogue accompanying the dermatologist's examination was particularly helpful in understanding what they were asked to do for the study."

DISCUSSION

20) CONSORT: Trial limitations, addressing sources of potential bias, imprecision, multiplicity of analyses
20-i) Typical limitations in ehealth trials
"Since the fiscal commitment to developing the Internet intervention was small, the Internet intervention had limited interactivity and personalization. The sample size was limited to one institution and the population was of a slightly higher socio-economic status (income of $51,000 and higher) than the national income average ($42,979), which may limit generalizability of our findings, especially in regards to prior experience with the tablet technology [9]"
"As the study has not finished the two year period of follow-up with the participating pairs, conclusions cannot be made about the accuracy of SSE as performed by the pairs."
21) CONSORT: Generalisability (external validity, applicability) of the trial findings
21-i) Generalizability to other populations
"The sample size was limited to one institution and the population was of a slightly higher socio-economic status (income of $51,000 and higher) than the national income average ($42,979), which may limit generalizability of our findings, especially in regards to prior experience with the tablet technology [9]"
21-ii) Discuss if there were elements in the RCT that would be different in a routine application setting
"This allowed the Internet intervention to be effective across a range of socio-economic classes. However, because the intervention required a secure Internet connection, the feasibility of using this tablet intervention to supplement the usual patient education depended on whether the doctor's office was able to provide Internet access. The Internet intervention was a method of teaching patients and their skin-check partner how to conduct monthly skin self-examinations more consistently than having a conversation with a healthcare provider (HCP), and could be provided at less cost than delivery by a HCP. A caveat is that much of the cost associated with the Internet intervention is incurred at the design and development stage rather than in delivering the intervention to the individual patient."
22) CONSORT: Interpretation consistent with results, balancing benefits and harms, and considering other relevant evidence
22-i) Restate study questions and summarize the answers suggested by the data, starting with primary outcomes and process outcomes (use)
"In this study, performance of skills of those participating in the Internet intervention group was worse than those receiving the in-person education, but better than the workbook group. While the in-person intervention was superior to the Internet intervention in both skills acquisition and promotion of self-efficacy, the advantage of the Internet intervention was the consistent delivery. The personalization of the Internet intervention afforded by the video of the physician and research coordinator working with patients could not be achieved via workbook or the in-person intervention. While the highest skills quiz scores were obtained by the in-person pairs, the pairs participating in the Internet intervention performed better than the pairs reading the workbook. This skills test performance suggested an Internet intervention can be more effective than passively reading information. In the future, a technologically more sophisticated and interactive Internet educational intervention may approach that of a face-to-face intervention in promoting self-efficacy and clarity of explanation of content."
22-ii) Highlight unanswered new questions, suggest future research
"In the future, this pilot Internet intervention will be redesigned to be more interactive, which is expected to improve the pairs' self-efficacy such that the intervention will be comparable with the in-person education."

Other information

23) CONSORT: Registration number and name of trial registry
Trial Registration: ClinicalTrials.gov NCT 01013844
24) CONSORT: Where the full trial protocol can be accessed, if available
The protocol can not yet be accessed as the study is not complete at this time.
25) CONSORT: Sources of funding and other support (such as supply of drugs), role of funders
Funding: Supported by 5R01 CA-154908 to June K. Robinson, MD, from the National Cancer Institute

The research was supported in part by resources provided by the Northwestern University Skin Disease Research Center (P30AR057216), Chicago, IL with support from NIH/NIAMS. Any opinions, findings, and conclusions or recommendations expressed in this material are those of the authors and do not necessarily reflect the view of the Northwestern University Skin Disease Research Center or the NIH/NIAMS.
X26-i) Comment on ethics committee approval
Not relevant to the aim of our paper.
x26-ii) Outline informed consent procedures
Not relevant to the aim of our paper.
X26-iii) Safety and security procedures
Not relevant to the aim of our paper.
X27-i) State the relation of the study team towards the system being evaluated
"Conflicts of Interest: None declared."
